# User characteristics of national smoking cessation services in Korea: who chooses each type of tobacco cessation program?

**DOI:** 10.1186/s12913-018-3817-z

**Published:** 2019-01-08

**Authors:** Bo Yoon Jeong, Min Kyung Lim, E. Hwa Yun, Jin-Kyoung Oh

**Affiliations:** 1 Division of Cancer Prevention, National Cancer Control Institute, National Cancer Center, Goyang, Republic of Korea; 2 Department of Cancer Control and Population Health, Graduate School of Cancer Science and Policy, National Cancer Center, Goyang, Republic of Korea

**Keywords:** Smokers, Cessation services, User characteristics, Nicotine dependency

## Abstract

**Background:**

Relatively little is known about which characteristics of smokers are related to choosing a specific type of smoking cessation service. The user characteristics of different smoking cessation services were compared to determine the service preferred by user characteristics.

**Methods:**

The characteristics of adult smokers from Korea National Health and Nutrition Examination Survey (3762) and registered users of national smoking cessation services operated through the web (14,762), at Public Health Center-based Smoking Cessation Clinics (PHC-based SCCs) (335,532), and by telephone (Quitline) (2983) were compared.

**Results:**

Females and younger aged were more in web and telephone-based cessation service users, while aged 50 years or older were more in PHC-based SCCs users. Although manufacturing and production workers were the most prevalent among smokers in the general population, office workers and others including housewives and unemployed were most prevalent among the users of Quitline and PHC-based SCCs, respectively. The number of cigarettes smoked per day was twice as high among cessation service users as in general population. Smokers with greater nicotine dependency were most prevalent in the web-based service. Overseas users were in the web-based cessation service.

**Conclusion:**

Identifying user-specific characteristics by the type of cessation services looks necessary to develop and offer appropriate cessation services.

## Background

Smoking is recognized as an addictive behavior that needs systematic help from society for discontinuation; thus, active intervention for smoking cessation is considered crucial [1–3]. Therefore, tobacco cessation services have been developed [4, 5] and offered in many countries [6]. Although the type of service differs among countries depending on the available resources, at least two types of cessation services are offered together to achieve user convenience and increase coverage in some countries [7–9]. Relatively little is known about which characteristics of smokers are related to choosing a specific type of service within the same society.

In the Republic of Korea, where the prevalence of smoking is high despite significant progress in reducing that rate from 28.8% in 2005 to 23.2% in 2013 and smoking-attributable mortality has been increased from 30.8% in 2003 to 34.7% in 2012 for male (from 5.78% in 2003 to 7.2% in 2012 for female), three types of smoking cessation services have been developed and adopted at the national level with governmental support. [10, 11] Thus, a large proportion (57.5%) of current Korean smokers has tried to quit, and PHC-based SCCs has been described as highly cost effective service. [12, 13].

However, there has been little cross-comparison of user characteristics of different types of cessation services, which is important to determine the most appropriate type of service for the target population and to make each service could focus more on appropriate target population.

In this context, the present study aimed to identify which type of smokers choose which type of cessation service as well as how the users of cessation services differ from smokers in the general population.

## Methods

### Overview of smoking cessation services in Korea

As one of its major efforts toward reducing cigarette smoking, the Korean government has implemented nationwide smoking cessation programs funded by cigarette taxes. A web-based smoking cessation service (WSCS), public health center (PHC)-based smoking cessation clinics (SCCs), and a telephone-based cessation service (Quitline) have been established in 2002, 2004, and 2006, respectively. These programs, which are grounded in the trans-theoretical model, have tailored protocols for adult men and women and adolescents, and they are open to all smokers for voluntary participation.

The WSCS offers a stand-alone program with 30 steps lasting 6 months that involves an interactive tool structured to respond to user smoking behaviors, needs for services, and completeness of stepwise counseling on the web. Users can get additional information or advice from experts by telephone or email.

SCCs offer both face-to-face counseling and nicotine replacement therapy (NRT) for 6 months after registration in the program. At least three face-to-face counseling sessions are required for registered users, and several additional telephone-based counseling sessions are offered during the program. This program is unique in its use of convenient community public health centers that are easily accessible to a large fraction of the Korean population, including those in rural areas. Around 700 trained coaches have worked to deliver cessation services at 250 PHC-based SCCs.

For registered users in Quitline, where 13 coaches have worked, a dedicated coach provides at least 21 protocol-based telephone counseling sessions for 1 year. Additionally, quit guide booklets are provided and periodic text messages are sent to users’ mobile phones, even if NRT and other types of pharmacotherapy are not available.

### Study participants

A secondary data from the result of each cessation service operation were used. An estimated 14,762, 335,532, and 2983 adult smokers aged more than 19 years who were registered users of a WSCS, PHC-SCCs, and Quitline, respectively, in 2009, when there was no distinct promotion of each cessation service, were included for a cross-comparison of user characteristics. A total of 3762 smokers among the 23,480 participants in the Korea National Health and Nutrition Examination Survey (KNHANES IV, 2007–2009) were included as a reference for the general characteristics of smokers in Korea. KNHANES is the national health survey has been done annually by Korean Center for Disease Control and could represent Korean population with the stratified multistage probability sampling method based on geographical area and housing type. It includes information on health behaviors, health examination results, food consumption, some of major disease prevalence, and etc. [14]

### Data and statistical analysis

From the basic user information collected to develop tailored counseling, common items were drawn for cross-comparison. Sex, age (19–29, 30–39, 40–49, and ≥ 50 years), area of residence (metropolitan, city/country, and overseas), average number of cigarettes smoked per day (≤10, 11–20, and ≥ 21), and nicotine dependency (based on the Fagerström Test for Nicotine Dependence: < 3, mild; 4–6, moderate; and 7–10, severe) [15] data were available for all three types of services. Information on occupation (professionals and experts, office workers, service workers, manufacturing and production workers, farming and fishing workers, small business owners, military and police, and others) was not available for WSCS participants. Comparable information, except for nicotine dependency, was available for smokers in the general population, represented by the data from KNHANES IV. The frequency distribution of these basic characteristics among smokers in the general population and users of each cessation service was presented and compared.

Various characteristics of users are presented by frequency distribution. The statistical significance test of each variable was guaranteed by chi-square test. The statistical tests were conducted using SAS (SAS Institute, Inc., Cary, North Carolina, USA), version 9.4 and *p* < .05 was considered statistically significant.

## Results

The proportion of female smokers was smaller among users of PHC-based SCCs (9.6%) than among users of the WSCS and Quitline as well as in the general population (Table 1). The proportion of smokers over the age of 50 was greatest among users of a PHC-based SCC (38.0%), while the proportions of smokers aged 19–29 and 30–39 were highest among the users of the WSCS (45.7%) and Quitline (40.6%), respectively. The distribution of smokers by area of residence was similar among users of all three types of services and in the general population, except that overseas users were identified only in the WSCS.

**Table 1:** Characteristics of smokers in the general population and users of three different national smoking cessation services in Korea

	Smokers from KNHANES IV	Smokers who used a Web-based cessation service	Smokers who used Quitline	Smokers who used a PHC-based SCC	*p*-value
	*n* = 3762	*n* = 14,762	*n* = 2983	*n* = 335,532	
Gender					
Male	3177 (87.2)	12,533 (84.9)	2610 (87.5)	303,220 (90.4)	<.0001
Female	585 (12.8)	2229 (15.1)	373 (12.5)	32,312 (9.6)	
Age group (years)					
19–29	640 (24.6)	6746 (45.7)	753 (25.2)	45,997 (13.7)	<.0001
30–39	945 (27.0)	5781 (39.2)	1210 (40.6)	82,430 (24.6)	
40–49	819 (23.2)	1794 (12.2)	731 (24.5)	79,779 (23.8)	
≥ 50	1358 (25.2)	441 (3.0)	289 (9.7)	127,326 (38.0)	
Area of residence					
Metropolitan	2119 (53.1)	8516 (57.7)	1639 (56.4)	195,956 (58.4)	<.0001
City/country	1643 (46.9)	6030 (40.9)	1268 (43.6)	139,576 (41.6)	
Overseas	–	216 (1.5)	–		
Occupation					
Professionals and experts	482 (14.7)	–	369 (12.4)	44,307 (13.2)	<.0001
Office workers	340 (9.6)	–	973 (32.8)	45,845 (13.7)	
Service workers	565 (16.7)	–	361 (12.2)	–	
Manufacturing and production workers	1060 (30.3)	–	125 (4.2)	22,955 (6.8)	
Farming and fishing workers	331 (4.9)	–	15 (0.5)	17,181 (5.1)	
Small business owners	–	–	396 (13.3)	72,634 (21.7)	
Military and police	14 (0.3)	–	73 (2.5)	14,694 (4.4)	
Others (e.g., housewives, retired or unemployed people, university students)	943 (23.4)	–	658 (22.2)	117,916 (35.1)	
Average number of cigarettes smoked per day					
≤ 10	1483 (38.8)	2034 (14.9)	788 (26.6)	71,836 (21.4)	<.0001
11–20	1831 (49.2)	7750 (56.6)	1524 (51.4)	177,958 (53.0)	
≥ 20	444 (12.0)	3899 (28.5)	651 (22.0)	85,738 (25.6)	
Nicotine dependency					
0–3 (mild)	–	3118 (24.2)	1290 (44.0)	105,121 (31.3)	<.0001
4–6 (moderate)	–	5584 (43.4)	1105 (37.7)	137,686 (41.0)	
7–10 (severe)	–	4180 (32.5)	537 (18.3)	92,725 (27.6)	

chi-square test

While manufacturing and production (30.3%) was the most prevalent occupational category among smokers in the general population, office workers were most prevalent among the users of Quitline (32.8%) and “others” (e.g., housewives, unemployed people, and university students) were the most prevalent among users of PHC-based SCCs (35.1%). These occupational categories were followed by “others” and small business owners, respectively.

The number of cigarettes smoked per day was about twice as high among service users as in the general population. Smokers who had greater nicotine dependency were most prevalent among users of the WSCS and least prevalent among the users of Quitline.

The characteristics of the users of the smoking cessation service were statistically significant according to the type of smoking cessation services (chi-square test, *p* < 0.001).

## Discussion

This study analyzed and compared the general characteristics of users who accessed three types of national smoking cessation services in one country to determine which smokers chose which type of cessation service. The user characteristics for the three types of smoking cessation services differed by sex, age, occupation, and level of nicotine dependency. They also differed from the characteristics of smokers in the general population. The cessation rate of each service is different or not directly comparable because service contents and form of delivery are different by the type of service. In addition, WSCS do not calculate and suggest the cessation rate, Quitline usually accounts smoking cessation rate as the 1-year abstinence rate (25.2% in 2009) which do not allow only one puff of cigarette smoke after starting quit, and SCCs suggested 6-month cessation rate (44.0% in 2009) which is not strict as much as abstinence rate in Quitline.

Based on our results, WSCS could be appropriate for younger smokers who are more familiar with using the internet and are more engaged in Internet-based social networks and activities than offline ones [16, 17]. As identified, WSCS could also be appropriate for overseas users. This information could be used to develop smoking cessation strategies for immigrants who come from countries where smoking is common and who therefore have different social and cultural contexts for smoking and cessation behaviors [18–20].

In contrast, Quitline could focus on middle-aged office workers who are less comfortable using a WSCS than younger smokers and who are engaged in their jobs during the daytime. These users could receive individualized support for cessation tailored to their own needs and daily schedules. A telephone-based service can be timed to maximize the level of support around a planned quit date and can be scheduled in response to the needs of the recipient and Quitline itself [21].

For older smokers (over the age of 50), housewives, and retired or unemployed people, who are the predominant users of free community-based health services, PHC-SCCs could be prioritized. These people might have enough time to visit a PHC- SCCs, have easy access to the services and expect emotional support via direct human contact. In particular, older smokers, those who have been smoking for a long time, and those who have previous experience quitting may feel it is little for them to learn from behavioral treatment, but their experience of past failures may fuel a perceived need for pharmacological treatment, including NRT [22, 23].

For female smokers, web- and telephone-based cessation services, which offer an anonymous form of service delivery, would be highly desirable because of the social stigma associated with female smoking in Korea. How manufacturing and production workers could be effectively reached by cessation services should be considered since most smokers in the general population are manufacturing and production workers, but most service users are not. Different levels of user nicotine dependency among users of the three types of cessation services should be considered carefully because smokers showing higher levels of nicotine dependency were more prevalent among users of the WSCS, which is a stand-alone program and therefore is not usually appropriate for highly addicted smokers.

Regarding the main study results mentioned above, differences on the familiarity on the service channel, accessibility on each service offered, a form of service delivery in terms of confidentiality, and availability of contents offered by each service might cause smokers to choose a different type of service. Although access and availability of each type of cessation service are not directly linked to the effectiveness of cessation, it is an important issue how much cessation service can reach to smokers without regard to user characteristics and service type deciding accessibility and availability of each cessation service. Furthermore, to achieve the effectiveness of cessation service delivery, it looks necessary to make smokers could access to the appropriate type of cessation service not only for their convenience and service accessibility but also for their smoking behaviors including nicotine dependency and cessation contents offered by each service.

Limited information on user characteristics was available for the comparison and information on occupation was not available for WSCS participants. As well, even though some of smokers might have experience to use more than one type of cessation services, it was not accounted in the comparison of user characteristics by the type cessation services. Therefore, it was not fully explained what factors associated with the difference of user characteristics in each service, even the differences in user characteristics shown in the present study. Those are the limitation of the present study due to using secondary data with the cross-sectional design. However, they could not affect the main result of the present study which is cessation service preferred might be different by the user characteristics and preference depending on the contents and accessibility of each cessation service offers.

Recently, more different type of cessation services, which are hospital-based cessation camp, cessation clinics supported by national health insurance cooperation, outreach program to help cessation for specific target population such as female, adolescents, and military serviceman, have been developed and launched at the national level to expand accessibility of smokers to cessation service. Therefore, in the future, repeating the comparison study including other service types newly launched could be expected. In addition, studies to clarify the reasons for the differences in user characteristics shown in the present study are needed. These results could be referred to by other countries where cessation services are planned or under development as well as they could provide practical feedback for improving national smoking cessation services in Korea in terms of how appropriate types of services could be suggested regarding user characteristics and preferences. Furthermore, offering specific type of cessation services regarding the characteristics or situation of smokers which is related to the accessibility to the services, easiness of service use, confidentiality on the privacy, and etc. could contribute to reduce the smoking prevalence, more effectively.

## Abbreviations

KNHANES: Korea National Health and Nutrition Examination Survey
NRT: Nicotine Replacement Therapy
PHC: Public Health Center
SCC: Smoking Cessation Clinic
